# Manualized group cognitive behavioral therapy for social anxiety in first-episode psychosis: a randomized controlled trial

**DOI:** 10.1017/S0033291721005328

**Published:** 2023-06

**Authors:** Martin Lepage, Christopher R. Bowie, Tina Montreuil, Larry Baer, Olivier Percie du Sert, Tania Lecomte, Ridha Joober, Amal Abdel-Baki, G. Eric Jarvis, Howard C. Margolese, Luigi De Benedictis, Norbert Schmitz, Ashok K. Malla

**Affiliations:** 1 Prevention and Early Intervention Program for Psychoses, Douglas Mental Health University Institute, Montreal, Quebec, Canada; 2 Department of Psychiatry, McGill University, Montreal, Quebec, Canada; 3 Department of Psychology, Department of Psychiatry, Centre for Neuroscience Studies, Queen's University, Kingston, Ontario, Canada; 4 Departments of Educational & Counselling Psychology and Psychiatry, McGill University, Montreal, Quebec, Canada; 5 Child Health and Human Development, Research Institute of the McGill University Health Centre, Montreal, Quebec, Canada; 6 Department of Psychology, University of Montréal, Montréal, Quebec, Canada; 7 Clinique JAP-Centre hospitalier de l'Université de Montréal (CHUM), Montréal, Québec, Canada; 8 Centre de recherche du Centre hospitalier de l'Université de Montréal CRCHUM, Montréal, Québec, Canada; 9 Département de psychiatrie et d'addictologie, Université de Montréal, Montréal, Québec, Canada; 10 First Episode Psychosis Program, Jewish General Hospital, Department of Psychiatry, McGill University, Montreal, Quebec, Canada; 11 Prevention and Early Intervention Program for Psychoses, McGill University Health Centre, Montreal, Quebec, Canada; 12 Department of Psychiatry, McGill University, Montreal, Quebec, Canada; 13 Connec-T Clinic (First Psychotic Episode and Early Intervention Program), Institut universitaire en santé mentale de Montréal, Montreal, Quebec, Canada; 14 Department of Psychiatry and Addictology, University of Montreal, Montreal, Quebec, Canada; 15 Department of Population-Based Medicine, Institute of Health Sciences, University Hospital Tuebingen, Tuebingen, Germany

**Keywords:** Cognitive behavioral therapy, cognitive remediation, first-episode psychosis, social anxiety

## Abstract

**Background:**

Social anxiety (SA), a prevalent comorbid condition in psychotic disorders with a negative impact on functioning, requires adequate intervention relatively early. Using a randomized controlled trial, we tested the efficacy of a group cognitive-behavioral therapy intervention for SA (CBT-SA) that we developed for youth who experienced the first episode of psychosis (FEP). For our primary outcome, we hypothesized that compared to the active control of group cognitive remediation (CR), the CBT-SA group would show a reduction in SA that would be maintained at 3- and 6-month follow-ups. For secondary outcomes, it was hypothesized that the CBT-SA group would show a reduction of positive and negative symptoms and improvements in recovery and functioning.

**Method:**

Ninety-six patients with an FEP and SA, recruited from five different FEP programs in the Montreal area, were randomized to 13 weekly group sessions of either CBT-SA or CR intervention.

**Results:**

Linear mixed models revealed that multiple measures of SA significantly reduced over time, but with no significant group differences. Positive and negative symptoms, as well as functioning improved over time, with negative symptoms and functioning exhibiting a greater reduction in the CBT-SA group.

**Conclusions:**

While SA decreased over time with both interventions, a positive effect of the CBT-SA intervention on measures of negative symptoms, functioning, and self-reported recovery at follow-up suggests that our intervention had a positive effect that extended beyond symptoms specific to SA.

ClinicalTrials.gov identifier: NCT02294409.

## Introduction

Social anxiety (SA) represents a persistent fear of being scrutinized and negatively evaluated during social interactions, which in turn is linked to cognitions of how this perceived anxiety will be revealed and interpreted by others (Clark & Wells, 1995). SA is a common comorbid condition in people who have recently experienced a first episode of psychosis (FEP) (Achim et al., 2013; Chudleigh et al., 2011; Lowengrub, Stryjer, Birger, & Iancu, 2015; McEnery, Lim, Tremain, Knowles, & Alvarez-Jimenez, 2019; Michail & Birchwood, 2009; Romm et al., 2011; Voges & Addington, 2005) and has a significant and negative impact on functioning, independent of psychosis, including academic and occupational achievement and importantly social interactions (Kessler, Chiu, Demler, Merikangas, & Walters, 2005; Michail & Birchwood, 2014; Michail, Birchwood, & Tait, 2017). Yet, there are few validated psychosocial interventions currently available that are specifically adapted to young people with SA in the context of FEP.

Detecting and intervening early for SA in FEP is particularly crucial for two reasons. First, the prevalence is substantial, being diagnosed in some studies in up to one-third of FEP cases (Michail & Birchwood, 2009). In addition, there is growing recognition that subthreshold SA symptoms are common in FEP (Birchwood et al., 2007; Michail & Birchwood, 2009). Second, a recent meta-analysis (McEnery et al., 2019) across stages of psychosis revealed that the presence of SA significantly reduces functioning and lowers the subjective quality of life. Hence, there is an important need to intervene early to alleviate comorbid SA symptoms in FEP and avoid disabling long-term consequences.

A few studies have examined the impact of psychosocial interventions for SA in psychosis. Kingsep, Nathan, and Castle (2003) and Halperin, Nathan, Drummond, and Castle (2000) independently developed cognitive-behavioral therapy (CBT) interventions for SA in psychosis, and both reported that the group setting intervention was effective. Their pioneering approaches suffered, however, from some limitations as Michail et al. (2017) suggest. Neither Kingsep's nor Halperin's studies considered the notion of illness-related stigma. Further, the limited number of sessions did not include social skills or assertiveness training, despite both having been shown to be quite effective in the treatment of SA (Baker & Edelmann, 2002). Both studies also had a small sample size, and the CBT intervention was compared to a waitlist control, leaving the possibility that any structured group activity could improve SA symptoms.

An adapted CBT intervention for psychosis must target factors that are uniquely associated with psychosis. This includes self-stigmatization and feelings of being engulfed by the illness (Konsztowicz & Lepage, 2019). Self-stigma, which involves the internalization of stereotypes and prejudices related to having a mental illness, is quite present in FEP (Chen et al., 2016). Several studies have shown that negative beliefs about the self and psychosis are related to the presence of SA (Gumley, O'Grady, Power, & Schwannauer, 2004). Birchwood et al. (2007), compared participants with an FEP with or without SA and observed that those with SA reported greater shame attached to having a psychotic illness. They also perceived their diagnosis as creating a separation between them and others, contributing to the belief that they had lower social status. This idea was further refined by Michail and Birchwood (2013) who determined that shame cognitions contributed significantly to social anxiety, with shame proneness and loss of social status being more elevated in those with social anxiety and psychosis compared to those with psychosis only.

Similarly, poor social skills, delusions including persecutory ideas, and cognitive deficits associated with psychosis that may impair literacy must be considered in designing an SA intervention in the context of psychosis. We have recently developed a psychosocial intervention for SA that integrates these different aspects, and our preliminary data from an uncontrolled evaluation revealed a significant decrease in SA symptoms and a significant improvement in positive and negative symptomology (Montreuil et al., 2016).

To test our intervention, we conducted a multi-site randomized controlled trial in five early psychosis clinics in Montréal. The primary objective was to compare the efficacy of our group CBT-SA intervention to an active control condition involving cognitive remediation (CR). We elected to compare to another intervention to control for spontaneous changes in symptoms of social anxiety, the effects of social exposures from being in a group-based intervention, and therapeutic commitment. We selected CR as it does not target symptoms of social anxiety, can be delivered in a group format with parameters similar to the treatment group and our group had experience with this approach [e.g. (Benoit, Harvey, Bherer, & Lepage, 2016)]. We hypothesized that compared to CR, individuals receiving the CBT-SA intervention would report a greater reduction in SA symptomatology as assessed with multiple measures and that these gains would be maintained at follow-up assessments (3- and 6-months). The secondary objective of the study was to measure the effect of this intervention on clinical and functional outcomes. We hypothesized that the CBT-SA group would show better clinical outcomes (i.e. a decrease in positive and negative symptom-severity) and better improvement in measures of functioning.

## Methods

### Study design and participants

This trial was registered on ClinicalTrials.gov (identifier: NCT02294409). In this RCT, the experimental group received CBT-SA plus standard FEP clinical care, and the active control group received CR and standard FEP clinical care. This trial involved five first-episode psychosis clinics affiliated with either McGill University or Université de Montréal; PEPP-Montréal, Douglas Institute; the First Episode Psychosis Program from the Jewish General Hospital (JGH); the First Episode Psychosis Program from the McGill University Health Centre (MUHC) from the McGill network; the *Clinique Jeunes Adultes Psychotiques* from the Centre hospitalier de l'Université de Montréal (CHUM); and the Clinique Connec-T from the Institut Universitaire de Santé Mentale de Montréal. These catchment-area specific FEP programs all provide a complete range of services for teens and young adults suffering from psychosis, including evaluation, prevention, treatment, hospitalization, outpatient services, rehabilitation, modified assertive community case management, and community follow-ups. The interventions took place sequentially on a rotational basis in one of three sites (Douglas, CHUM or JGH), with participants attending from any of the five designated clinics which provided enough participants for randomization to the two groups while providing geographical diversity. Ten cohorts of CBT-SA and CR took place from Fall 2014 to Summer 2019, including six at the Douglas, two at CHUM, and two at the JGH. This project was approved by the research and ethics boards of all participating sites.

Participants within the first 2 years of treatment following the onset of a psychotic disorder were recruited from these programs. Inclusion criteria were: a diagnosis of a psychotic disorder; aged 18–35; ability to read and write English or French at an intermediate level (Education >8 years); scores above predetermined cut-offs on the three measures of SA [34 for the Social Interaction Anxiety Scale (Mattick & Clarke, 1998), 20 for the Social Phobia Inventory (Connor et al., 2000) and 21 for the Brief Social Phobia Scale (Davidson et al., 1991)]; and meet criteria for the diagnosis of SA disorder as determined with the SCID social phobia module. These scales are described further in the Primary Outcomes section. Exclusion criteria comprised: currently clinically unstable, defined as the presence of positive symptoms that are moderate to severe on the SAPS rating scale; IQ < 70; hospitalized at the time of recruitment; current diagnosis of substance dependence; lifetime history of a neurological condition.

### Interventions

#### Experimental intervention – Group Cognitive Behavioral Therapy for SA (CBT-SA)

All CBT-SA sessions were conducted in either English or French by trained clinicians with the aid of a group CBT-SA manual that is described in Montreuil et al. (2016). This intervention was delivered by a therapist (doctoral-level psychologist) and a co-therapist under the supervision of an experienced CBT therapist (M.L.). This intervention included the five following modules: (i) Psychoeducation on SA disorder, stress, psychosis and self-stigma; (ii) Cognitive restructuring: identifying negative thoughts that occur before, during, or after anxiety-provoking situations; (iii) Social Skills training, which provided an opportunity to practice interpersonal skills; (iv) Exposure component, which focused on the collection of information that would allow patients to revise their judgments about the degree of risk to which they are exposed in feared situations, and would challenge their dysfunctional beliefs; and (v) Relapse prevention and maintenance. Each of the 13 group sessions consisted of 4–8 participants and lasted for 1.5 h each. One session per week was provided for the 13-week period.

#### Active comparator – Computer-Assisted Cognitive Remediation Group Therapy; CR

This group intervention developed by Bowie, McGurk, Mausbach, Patterson, and Harvey (2012) devotes approximately 60% of the session to cognitive training activities on a computer, 20% to documenting and attempting new strategies for solving problems (‘strategic monitoring’), and 20% to doing ‘transfer activities’, which consist of simulated real-world activities and discussion of how cognitive strategies might be applied in everyday life and potential compensatory strategies for overcoming cognitive challenges. This approach specifically targets processing speed, attention, memory, and executive function because they are domains that are commonly impaired in psychosis. The parameters of this intervention were closely matched to that of the group CBT-SA intervention and involved 13 weekly sessions of 1.5 h. The software Brain Training Pro (www.scientificbraintrainingpro.fr) was used. Each 1.5-h session was conducted with groups of 4–8 participants and started with 60 min of supervised individual training with computer activities and included therapist prompts for patients to monitor and document their cognitive strategies. Each session ended with the whole group participating in transfer activities led by the therapist and co-therapist. The content of these activities was modified from the original tasks in that the interactive role-plays were removed and others were modified to reduce social interactions in order to serve as a control for the SA treatment.

### Randomization and masking

Random assignment was carried out using a computer-run randomization protocol by a biostatistician not associated with treatment in any capacity and located away from the treatment sites. Recruitment of participants and symptom assessments were conducted, respectively, by a research coordinator and a research assistant, and the latter was blind to treatment assignment. Across the five different institutions, 210 potential participants were referred and screened. We recruited 96 individuals (51 randomized to group CBT-SA; 45 randomized to CACRT) who were relatively clinically stable with regards to their psychotic symptoms.

### Outcome measures

#### Primary outcomes

The primary outcome measures consisted of three complementary measures of SA. The Social Interaction Anxiety Scale, SIAS (Mattick & Clarke, 1998) is a 20-item instrument that measures anxiety in interpersonal encounters; the Social Phobia Inventory, SPIN (Connor et al., 2000) is a 17-item scale assessing multiple dimensions of SA including fear, avoidance and physiological discomfort; the Brief Social Phobia Scale, BSPS (Davidson et al., 1991) is an 11-item clinician-rated assessment which measures fear, avoidance and autonomic physiological responses to common social situations. Total scores were then standardized and averaged to create a composite score of SA.

#### Secondary outcomes

The secondary outcomes were comprised of clinical and functional measures. For clinical outcomes, changes in positive and negative symptom severity from pre-treatment to follow-ups were measured. For assessment of positive and negative symptoms, we used the Scale for Assessment of Positive Symptoms, SAPS (Andreasen, 1984a) and the Scale for Assessment of Negative Symptoms, SANS (Andreasen, 1984b). The Recovery Assessment Scale, RAS (Giffort, Schmook, Woody, Vollendorf, & Gervain, 1995) and the Social and Occupational Functioning Scale, SOFAS (Morosini, Magliano, Brambilla, Ugolini, & Pioli, 2000) were used to assess complementary aspects of functioning. The RAS is a 41-item self-report measure examining multiple dimensions of subjective functional recovery. The SOFAS is used to evaluate the individual's level of social and occupational functioning independent of psychiatric symptoms.

#### Other measures

Participants who met the inclusion criteria and signed informed consent were administered a clinical assessment at intake and subsequently repeated at post-therapy, 3-month and 6-month follow-ups. In addition to positive and negative symptoms, depression was assessed with the Calgary Depression Scale, CDS (Addington, Addington, & Schissel, 1990). The Internalized Stigma of Mental Illness, ISMI (Ritsher, Otilingam, & Grajales, 2003) a self-report questionnaire, was used to measure the subjective experience of stigma. Higher scores indicate higher internalized stigma. Initial assessment of participants also included the WASI (Wechsler, 1999), a brief IQ test. Participants were also evaluated using the CogState (Pietrzak et al., 2009) computerized battery involving 11 tasks covering seven cognitive domains.

### Statistical analysis

Descriptive statistics were examined to ensure that data met statistical test assumptions. Baseline sociodemographic characteristics were compared using independent-samples *t* tests or χ^2^, to examine between-group differences and assess the randomization procedure's effectiveness. We similarly examined such differences between completers and non-completers for the two interventions. The primary and secondary outcome analyses were pragmatic, based on intention to treat (analyzed by assigned treatment group after randomization regardless of the length of treatment received), and utilized all available follow-up data from randomized participants. For primary outcome analyses, we used linear mixed models (LMM) for repeated measures over time to examine the impact of the two group interventions on a composite score of SA at baseline and follow-up with fixed effects of time, group, and the interactions between time and group with linear trends. Age, sex, and baseline score were used as covariates while the number of sessions completed, and the severity of positive symptoms was further controlled in exploratory analyses reported in the online Supplementary data. As multiple imputations are not recommended if a large proportion of data are missing (Jakobsen, Gluud, Wetterslev, & Winkel, 2017), we used the maximum-likelihood method to handle missing data which prevented listwise deletion due to such missing data.

## Results

### Participants

**Fig. 1:**
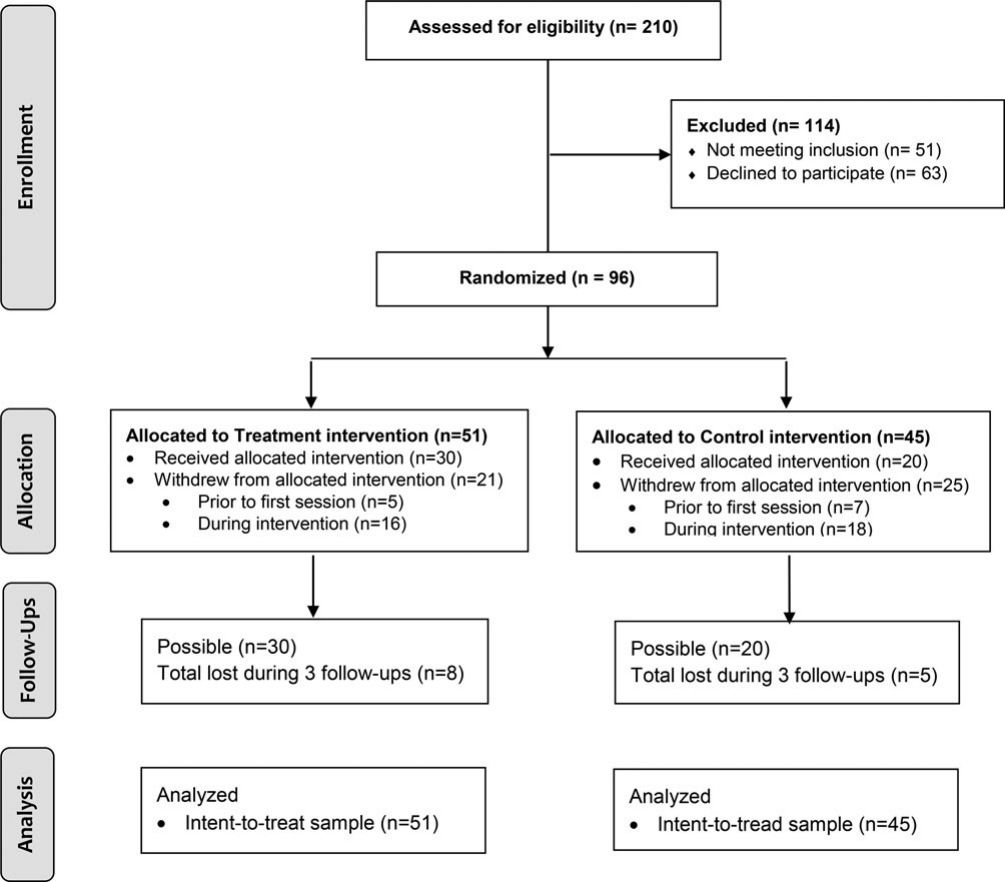
CONSORT diagram for the randomized controlled trial.

Figure 1 illustrates the participants' enrollment, randomization, and flow. Table 1 presents the sociodemographic variables for both groups and comparisons between groups. Between-group comparisons did not reveal any significant differences in baseline characteristics. Potential adverse effects associated with participation in this trial were monitored via self-reports. Three serious adverse effects were reported and consisted of hospitalizations for two participants (one was hospitalized twice). These two participants were randomized to the CBT intervention but there was no connection between participation in our intervention and these hospitalizations.

**Table 1.** Sociodemographic and clinical characteristics at baseline by treatment group^a^

Characteristic	CBT-SA (*n* = 51)		CR (*n* = 45)	
	*n*	%	*n*	%
Sex (F/M)	20/31	39/61	13/32	29/71
Language (English/French/other)	21/26/4	41/51/8	18/22/5	40/49/11
SES^b^				
Lower	5	9.8	4	8.9
Lower middle	10	19.6	7	15.6
Middle	16	31.4	9	20.0
Upper middle	9	17.6	8	17.8
Upper	4	7.8	8	17.8
Unknown	7	13.7	9	20.0
Diagnosis				
Schizophrenia	10	19.6	11	24.4
Schizophreniform disorder	7	13.7	7	15.6
Schizoaffective disorder	3	5.9	4	8.9
Delusional disorder	1	2	0	0
Brief psychotic disorder	2	3.9	1	2.2
Substance-induced psychotic disorder	3	5.9	1	2.2
Psychotic disorder NOS	14	27.5	13	28.9
Major depression with psychotic features	4	7.8	3	6.7
Bipolar disorder with psychotic features	7	13.7	5	11.1
Site				
Douglas Institute	19	37	20	44
Jewish General Hospital	13	26	11	24
CHUM	12	24	11	24
MUHC	5	10	3	7
IUSMM	2	4	0	0
	*M*	s.d.	*M*	s.d.
Age (years)	25.33	4.39	23.82	4.36
Education (years)	12.14	2.13	11.73	2.27
IQ (WASI)	102.31	11.70	100.73	12.68
Duration of illness (years)	1.56	1.46	1.44	1.50
# of hospitalizations	1.00	1.20	1.00	1.33
Duration of hospitalizations (days)^c^	13.21	28.40	20.17	29.25
Antipsychotic dose in chlorpromazine equivalents	196	210	221	223

a There were no significant differences on any variables between groups.

b Socioeconomic status was rated using the Hollingshead two-factor index of social position with modification of the education scale for Quebec.

c Value refers to the grand mean of each participant's mean length of stay.

### Acceptability

Of the 96 randomized participants, 46 (48%) dropped out from the study before completing the intervention. Online Supplementary Table S1 provides a comparison between completers and non-completers. As can be observed, non-completers were significantly younger, had fewer years of education and an earlier age at the onset of psychosis. Measures of symptoms revealed no significant differences between these two groups except for the SIAS on which non-completers had significantly lower scores. A greater percentage of participants randomized to CR withdrew from the intervention (56%) relative to those randomized to CBT-SA (41%) but this difference was not significant [odds ratio = 0.56; 95% CI (0.25–1.26); *p* = 0.159].

### Primary outcome – measures of social anxiety

Primary outcomes consisted of three standardized measures of SA (SIAS, SPIN and BSPS) which were first combined into a composite measure to provide an omnibus test and then were further examined separately. LMM analyses revealed that both randomized groups exhibited significant improvement overtime on the composite score of SA [*F*(3, 168.27) = 40.28, *p* < 0.001] and no significant interaction (*p* = 0.832) nor group differences (*p* = 0.912) were observed. In addition, none of the covariates was significant except for the severity of SA at baseline [*F*(1, 124.62) = 216.14, *p* < 0.001]. Further covarying for positive and negative symptoms and a number of sessions did not significantly change our results. Table 2 presents comparisons between the two groups at each time point. Separate analyses of all three measures of SA provided similar results and were reported in online Supplementary Table S2.

**Table 2.** Primary and secondary outcomes as a function of group and timepoint^a^

	CBT-SA			CR			Mean difference (95% CI)	*p* Value
	Mean	s.d.	*n*	Mean	s.d.	*n*		
Primary outcome – composite SA score								
Pre-therapy	54.07	10.67	49	53.87	11.25	43	0.21 (−4.24 to 4.65)	0.928
Post-therapy	40.94	13.11	29	40.85	14.89	19	0.09 (−5.66 to 5.84)	0.976
3-month f-u^b^	41.54	14.62	25	39.76	16.81	16	1.78 (−4.67 to 8.22)	0.587
6-month f-u	35.94	15.78	22	37.06	17.71	15	−1.12 (−7.98 to 5.74)	0.748
Secondary outcomes								
SAPS								
Pre-therapy	11.89	6.22	50	12.46	6.49	43	−0.57 (−3.13 to 2)	0.664
Post-therapy	9.06	8.00	29	8.67	9.09	19	0.39 (−3.1 to 3.89)	0.826
3-month f-ub	10.64	8.80	25	9.26	10.09	16	1.39 (−2.47 to 5.24)	0.480
6-month f-u	7.57	9.43	22	7.10	10.48	15	0.48 (−3.59 to 4.54)	0.818
SANS								
Pre-therapy	23.83	6.31	50	23.8	6.54	43	0.03 (−2.57 to 2.63)	0.980
Post-therapy	26.04	8.24	29	29.44	9.37	19	−3.40 (−7.01 to 0.21)	0.065
3-month f-u	20.14	9.00	25	25.78	10.30	16	−5.64 (−9.59 to −1.68)	0.005*
6-month f-u	18.86	9.64	22	25.07	10.66	15	−6.20 (−10.36 to −2.05)	0.004*
RAS								
Pre-therapy	153.68	14.79	50	153.88	15.25	43	1.35 (−7.6 to 10.3)	0.766
Post-therapy	165.24	17.67	29	160.19	20.37	19	7.51 (−3 to 18.02)	0.160
3-month f-u	166.63	19.85	25	163.94	23.10	16	6.66 (−5.16 to 18.48)	0.267
6-month f-u	171.94	21.22	22	163.88	24.35	15	13.01 (0.26 to 25.75)	0.046*
SOFAS								
Pre-therapy	51.85	7.76	50	52.15	8.03	43	−0.30 (−3.49 to 2.89)	0.852
Post-therapy	54.65	9.91	29	51.06	11.25	19	3.59 (−0.74 to 7.92)	0.103
3-month f-u	58.75	10.88	25	53.48	12.47	16	5.27 (0.49 to 10.04)	0.031*
6-month f-u	60.29	11.67	22	56.72	12.96	15	3.57 (−1.47 to 8.61)	0.164

SAPS, Scale for the Assessment of Positive Symptoms total (composite) score was calculated by summing all items except for the global rating items; SANS, Scale for the Assessment of Negative Symptoms total (composite) score was calculated by summing all items except for the global rating items; RAS Recovery Assessment Scale, SOFAS, Social and Occupational Functioning Scale.

a Estimated means are presented in this table.

b f-u: follow-up.

*Significant *p* value <0.05.

### Secondary outcomes – clinical and functional outcomes

LMM analyses on SAPS revealed a significant effect of time [*F*(3174.80) = 7.66, *p* < 0.001], no significant effect of the group nor a significant time × group interaction (all *p*'s > 0.71). For SANS, a significant effect of time [*F*(3189.33) = 8.46, *p* < 0.001], group [*F*(1110.39) = 11.91, *p* < 0.001] and a significant time × group interaction [F(3189.46) = 2.94, *p* < 0.04]. Groups significantly differed at 3-month (*p* < 0.005, Cohen's *d* = 0.57) and 6-month (*p* < 0.004, Cohen's *d* = 0.60) follow-ups with the CBT-SA group exhibit lower negative symptoms severity relative to the CR group. Only sex was a significant covariate [*F*(1129.91) = 8.37, *p* < 0.005] with males exhibiting higher negative symptom severity than females. On the SOFAS, only a significant effect of time [*F*(3178.51) = 7.00, *p* < 0.001], and of a group [*F*(1108.69) = 4.56, *p* < 0.04] were revealed. On the RAS, only a significant effect of time [*F*(3161.65) = 12.50, *p* < 0.001] was observed. Groups significantly differed on the SOFAS at 3-month (*p* < 0.03, Cohen's *d* = 0.44) and on the RAS at 6-month (*p* < 0.05, Cohen's *d* = 0.56) follow-ups with greater improvement in the CBT-SA group. Table 2 presents comparisons between the two groups at each time point for the primary and secondary outcomes.

For the RAS we further examined the 6-month follow-up of the five subscales (personal confidence and hope, willingness to ask for help, goal and success orientation, reliance on others, and not being dominated by symptoms) and only two significantly differed between the groups [willingness to ask for help; *t*(35) = −3022, *p* < 0.005, Cohen's *d* = 1.02 and reliance on others; *t*(35) = −2703, *p* < 0.01, Cohen's *d* = 1.01] with greater improvement in the CBT-SA group.

### Other outcome measures

Explorations of the CDS revealed only a significant effect of time [*F*(3183.00) = 5.66, *p* < 0.001]. On the ISMI, a significant effect of time [*F*(3169.71) = 15.65, *p* < 001], group [*F*(1115.91) = 4.78, *p* < 0.03] and a significant time × group interaction [*F*(3170.00) = 3.14, *p* < 0.03]. Groups significantly differed on the ISMI at 6-month (*p* < 0.004, Cohen's *d* = 0.60) follow-up. Finally, no significant effects were observed on the amount of prescribed antipsychotic. Table 3 presents comparisons between the two groups at each time point.

**Table 3.** Other outcome measures as a function of group and timepoint^a^

	CBT-SA			CR			Mean difference (95% CI)	*p* Value
	Mean	s.d.	*n*	Mean	s.d.	*n*		
CDS								
Pre-therapy	5.78	2.81	49	5.71	2.94	43	0.07 (−1.1 to 1.24)	0.906
Post-therapy	4.72	3.61	29	4.05	4.13	19	0.67 (−0.92 to 2.26)	0.409
3-month f-u^b^	4.73	3.96	25	4.53	4.68	15	0.2 (−1.58 to 1.98)	0.827
6-month f-u	3.99	4.24	22	3.65	4.74	15	0.34 (−1.5 to 2.18)	0.716
ISMI								
Pre-therapy	2.24	0.32	47	2.23	0.33	42	0.01 (−0.12 to 0.14)	0.886
Post-therapy	1.94	0.38	29	2.09	0.44	19	−0.15 (−0.33 to 0.02)	0.080
3-month f-u	1.93	0.43	25	2.03	0.49	16	−0.10 (−0.29 to 0.09)	0.305
6-month f-u	1.73	0.46	22	2.03	0.52	15	−0.30 (−0.5 to −0.1)	0.004*
CPZeq (mg)								
Pre-therapy	209.79	170.54	51	204.33	178.09	44	5.46 (−63.75 to 74.67)	0.877
Post-therapy	210.11	213.15	29	268.70	241.86	19	−58.59 (−150.41 to 33.22)	0.210
3-month f-u	206.82	237.47	25	286.50	272.70	16	−79.68 (−182.71 to 23.35)	0.129
6-month f-u	248.24	256.29	22	309.82	286.25	15	−61.59 (−171.10 to 47.93)	0.269

CDS, Calgary Depression Scale; ISMI, Internalized Stigma of Mental Illness; CPZeq, Antipsychotic dose in chlorpromazine equivalents.

a Estimated means are presented in this table.

b f-u : follow-up.

*Significant *p* value <0.05.

## Discussion

This study examined whether a CBT group intervention, designed to treat SA in the context of an FEP, compared to CR was effective in improving symptoms of SA in addition to clinical and functional outcomes. Significant improvements on measures of SA over time for both intervention groups were observed. Positive and negative symptoms significantly improved over time, but the effect on negative symptoms was stronger at both follow-up time points in the CBT-SA group. Both measures of functioning (SOFAS and RAS) were associated with a significant improvement over time in both groups. Again, the CBT-SA group exhibited superior ratings on SOFAS at 3 month and on the RAS at 6-month follow-up relative to the CR group. Other outcomes were explored and notably, a decrease in self-stigmatization in the CBT-SA group at 6-month follow-up was noted. Taken together, these results suggest that while CBT-SA and CR had similar positive effects on symptoms of SA, the former had an additional and unique impact on negative symptoms and functioning while decreasing self-stigma.

### Social anxiety

We used three complementary measures of SA including two self-rated (SPIN, SIAS) and one clinician-rated (BSPS), yet all measures led to a similar pattern of results. We observed a significant reduction in SA severity in both intervention groups and this effect was most apparent from pre- to post-intervention. It could be the case that CR had some active ingredients for social anxiety. Notably, the interaction between patients during CR may have provided a form of exposure to social situations. This pattern is reminiscent of the findings of two large multi-site trials on novel CBT interventions and for which CR was used as a comparator. Klingberg et al. (2011) compared a CBT intervention for negative symptoms in 198 schizophrenia participants and observed similar improvement in both CBT and CR intervention. More recently, Pijnenborg et al. (2019) compared a group cognitive intervention to improve insight to CR in 121 persons with schizophrenia and similarly observed improvement in both conditions. Hence, such a significant impact of CR on symptomatology points to the challenge of devising an active control condition in behavioral interventions (Freedland et al., 2019).

Another possibility is that FEP is associated with elevated levels of SA early in the course of illness that progressively abate over time. As such, one would expect a steady decrease from pre-therapy to 6-month follow-up but most of the improvement was observed at post-therapy. A waitlist control comparator would have been necessary to confirm or disconfirm such a possibility.

### Negative symptoms and functioning

Negative symptoms improved significantly over time in both groups with greater improvement observed in the CBT-SA group at the two follow-up time points. This is an important finding as negative symptoms have been systematically related to poorer functioning in FEP and are often resistant to medication treatments (Bucci et al., 2020; Lepage et al., 2021). A previous meta-analysis similarly revealed a positive impact of CR on negative symptoms severity (Cella, Preti, Edwards, Dow, & Wykes, 2017). Our CBT-SA intervention exerted an influence that went over and beyond that of CR on negative symptom improvements. This effect was more pronounced at follow-up, an outcome that echoes the findings of another meta-analysis on psychological interventions for negative symptoms, which reported stronger effects over time (Lutgens, Gariepy, & Malla, 2017). While we can only speculate on the potential active ingredients in our CBT-SA intervention that led to improvements in negative symptoms, we suggest that cognitive restructuring and exposure may have enhanced motivation for social activities. In addition, sex was a significant covariate for negative symptoms with males exhibiting greater severity of negative symptoms than females, a finding consistent with previous studies (Buck et al., 2020).

Improvements in negative symptoms co-occurred with an increase in functioning as determined by ratings on the SOFAS for both intervention groups. This is consistent with recent meta-analyses of CR in psychosis which revealed a significant increase in functioning (Lejeune, Northrop, & Kurtz, 2021; Vita et al., 2021). Our two intervention groups nonetheless differed significantly at 3-month follow-up favoring CBT-SA, but this effect was no longer apparent at the 6-month follow-up. For the CBT-SA group, we observed a 10-point improvement on the SOFAS from pre-therapy to 6-month follow-up, whereas the improvement was relatively smaller in CR (3-point improvement). The focus of our CBT-SA intervention on social skills training and exposure work, which represented a significant proportion of this intervention (nearly 40% of sessions), may account for this effect.

### Recovery and self-stigma

Self-rated recovery improved in both treatment groups although at 6 months the CBT-SA group intervention showed superiority. A previous factor analysis of the RAS identified five factors (personal confidence and hope, willingness to ask for help, goal and success orientation, reliance on others, and not being dominated by symptoms) (Corrigan, Salzer, Ralph, Sangster, & Keck, 2004). Post hoc analyses on these factors revealed that willingness to ask for help and reliance on others, was significantly greater in the CBT-SA group. This is in line with the active ingredients of our CBT-SA intervention that targeted such social interactions.

Consistent with this improvement in self-reported recovery, the two intervention groups also significantly differed at 6-month follow-up on a measure of self-stigma (ISMI) with the CBT-SA group showing a greater reduction. Other studies have tested the efficacy of novel interventions targeting self-stigma (Best, Grossman, Milanovic, Renaud, & Bowie, 2018; Morrison et al., 2016; Young, 2018) or recovery of personal identity (Konsztowicz, Gelencser, Otis, Schmitz, & Lepage, 2021) and revealed the malleability of self-stigma. It is quite possible that the introduction of a psychoeducation module early in our intervention that covered psychosis and stigma provided the foundation for this outcome. In an exhaustive review of the literature of the RAS, this recovery measure was found to be associated with improved function and lower self-stigma (Salzer & Brusilovskiy, 2014) and our findings are consistent with these observations.

### Limitations

This study has several limitations that need to be taken into consideration. First, we observed a relatively high attrition rate, which nonetheless is within the range observed in a meta-analysis of psychosocial interventions for schizophrenia (Szymczynska, Walsh, Greenberg, & Priebe, 2017) and group therapy for social anxiety in non-psychotic participants (Hofmann & Suvak, 2006). This high attrition was, however, at variance with our prior feasibility study on 29 participants that reported a 10% attrition rate (Montreuil et al., 2016). We suspect that the randomization process and the requirement for many participants to attend another mental health institution to receive this intervention negatively impacted engagement. To partially compensate for this, our statistical analysis involving LMM allowed us to use all available data on those who dropped out. Second, we did not stratify for sex during randomization and had more women in the CBT group than then CR group. This could have influenced some of our results notably for negative symptoms. Third, the design of our study which involved an active comparator intervention, namely CR, may have obscured the efficacy of our CBT-SA intervention. The recently proposed Pragmatic Model for Comparator Selection in Health-Related Behavioral Trials (Freedland et al., 2019), provides interesting guidelines for the selection of an appropriate comparator as a function of the main research question. Given that the purpose of our trial was to establish the efficacy of our CBT-SA intervention, treatment as usual or wait-list condition might have been more appropriate.

## Conclusions

Our RCT revealed that our CBT-SA intervention, designed for young people with the FEP, improved symptoms of SA, but this intervention was not superior to an active control treatment (CR). A superior effect of the CBT-SA intervention on measures of negative symptoms, functioning, recovery, and stigma at follow-up suggests that the benefits of our experimental intervention extended beyond SA. The latter is clearly an important target of psychosocial interventions in early psychosis as improvement in negative symptoms will likely translate into better functioning, reduced internalized stigma, and a stronger sense of recovery.

## Supplementary material

For supplementary material accompanying this paper visit https://doi.org/10.1017/S0033291721005328. click here to view supplementary material
