# Supplementary material for: Manualized group cognitive behavioral therapy for social anxiety in first-episode psychosis: a randomized controlled trial
Source: Psychol Med. 2022 Jan 11;53(8):3335–44. doi: 10.1017/S0033291721005328 (PMC10277720; doi:10.1017/S0033291721005328)
Supplement: Supplementary file 1 [file S0033291721005328sup001.docx]

Manualized group cognitive behavioral therapy for social anxiety in first episode psychosis: A randomized controlled trial

**Supplemental data**

**Table S1.** Sociodemographic and clinical characteristics at baseline as a function of completers and non-completers.

| Variables | Completers | | Non-Completers | |  |  |
| --- | --- | --- | --- | --- | --- | --- |
|  | Mean/n | SD/% | Mean/n | SD/% | Test statistic | p |
| Sex |  |  |  |  | 1.88 | 0.170 |
| male | 36 | 72 | 27 | 58.70 |  |  |
| Female | 14 | 28 | 19 | 41.30 |  |  |
| Language |  |  |  |  | 0.94 | 0.624 |
| English | 19 | 38 | 20 | 43.50 |  |  |
| French | 25 | 50 | 23 | 50.00 |  |  |
| Bilingual | 6 | 12 | 3 | 6.50 |  |  |
| Diagnosis |  |  |  |  | 0.34 | 0.951 |
| Affective spectrum | 11 | 22 | 8 | 17.40 |  |  |
| Schizophrenia spectrum | 23 | 46 | 23 | 50.00 |  |  |
| Substance-induced | 14 | 28 | 13 | 28.30 |  |  |
| Not otherwise specified | 2 | 4 | 2 | 4.30 |  |  |
| Site |  |  |  |  | 11.67 | 0.009* |
| CHUM | 13 | 26 | 10 | 21.70 |  |  |
| Douglas | 16 | 32 | 23 | 50.00 |  |  |
| IUSMM | 2 | 4 | 0 | 0.00 |  |  |
| Jewish | 11 | 22 | 13 | 28.30 |  |  |
| MUHC* | 8 | 16 | 0 | 0.00 |  |  |
|  |  |  |  |  |  |  |
| Age (years) | 25.80 | 4.70 | 23.30 | 3.80 | 2.84 | 0.006* |
| Education (years) | 12.50 | 2.00 | 11.30 | 2.30 | 2.65 | 0.009* |
| IQ | 102.20 | 12.70 | 100.80 | 11.60 | 0.53 | 0.597 |
| SES | 37.40 | 18.90 | 40.80 | 16.70 | -0.85 | 0.396 |
| Duration of illness (years) | 1.40 | 1.30 | 1.70 | 1.60 | -1.01 | 0.317 |
| # of hospitalizations | 0.70 | 0.90 | 1.30 | 1.50 | -2.28 | 0.026* |
| Duration of hospitalizations (days) | 16.30 | 34.00 | 16.60 | 22.40 | -0.05 | 0.962 |
| Chlorpromazine equivalent (mg) | 230 | 238 | 184 | 187 | 1.05 | 0.298 |
| Social anxiety measures |  |  |  |  |  |  |
| SIAS | 48.40 | 13.50 | 40.80 | 12.90 | 0.94 | 0.348 |
| SPIN | 37.50 | 12.90 | 35.00 | 12.30 | 2.77 | 0.212 |
| BSPS | 37.30 | 13.40 | 31.80 | 12.60 | 2.03 | 0.046 |
| Other symptom measures |  |  |  |  |  |  |
| SAPS | 11.60 | 11.90 | 13.60 | 12.90 | -0.76 | 0.450 |
| SANS | 24.00 | 10.80 | 25.20 | 10.30 | -0.53 | 0.597 |
| CDS | 5.70 | 4.00 | 5.90 | 3.40 | -0.32 | 0.751 |
| Functioning |  |  |  |  |  |  |
| RAS | 151.50 | 21.00 | 156.60 | 19.70 | -1.15 | 0.253 |
| SOFAS | 51.50 | 12.10 | 51.20 | 10.40 | 0.13 | 0.898 |

* significant P value < 0.05

SIAS, Social Interaction Anxiety Scale; SPIN, Social Phobia Inventory; BSPS, Brief Social Phobia Scale; SAPS, Scale for the Assessment of Positive Symptoms total (composite) score was calculated by summing all items except for the global rating items; SANS, Scale for the Assessment of Negative Symptoms total (composite) score was calculated by summing all items except for the global rating items; RAS Recovery Assessment Scale, SOFAS, Social and Occupational Functioning Scale. CDS, Calgary Depression Scale.

**Table S2** Primary outcome measures as a function of group and timepoint^a^.

|  | CBT-SA | | | CR | | | Mean difference [95% CI] | p value |
| --- | --- | --- | --- | --- | --- | --- | --- | --- |
|  | Mean | SD | n | Mean | SD | n |  |  |
| **SIAS** |  |  |  |  |  |  |  |  |
| Pre-therapy | 47.12 | 9.33 | 50 | 46.89 | 9.78 | 43 | 0.23 [-3.61, 4.07] | 0.905 |
| Post-therapy | 38.31 | 11.65 | 29 | 37.53 | 13.42 | 19 | 0.78 [-4.35, 5.9] | 0.766 |
| 3-month f-u^b^ | 38.72 | 12.97 | 25 | 37.22 | 15.21 | 16 | 1.50 [-4.26, 7.26] | 0.609 |
| 6-month f-u | 32.65 | 13.97 | 22 | 33.68 | 16.03 | 15 | -1.03 [-7.16, 5.09] | 0.740 |
|  |  |  |  |  |  |  |  |  |
| **SPIN** |  |  |  |  |  |  |  |  |
| Pre-therapy | 36.16 | 7.95 | 50 | 35.97 | 8.35 | 43 | 0.20 [-3.09, 3.49] | 0.906 |
| Post-therapy | 26.50 | 9.99 | 29 | 24.16 | 11.24 | 19 | 2.34 [-2, 6.68] | 0.290 |
| 3-month f-u | 27.32 | 11.11 | 25 | 23.69 | 12.62 | 16 | 3.63 [-1.22, 8.48] | 0.142 |
| 6-month f-u | 21.97 | 11.96 | 22 | 21.8 | 13.23 | 15 | 0.17 [-4.97, 5.31] | 0.948 |
|  |  |  |  |  |  |  |  |  |
| **BSP** |  |  |  |  |  |  |  |  |
| Pre-therapy | 36.62 | 8.70 | 49 | 37.11 | 9.12 | 44 | -0.49 [-4.08, 3.1] | 0.786 |
| Post-therapy | 26.54 | 10.80 | 29 | 28.04 | 12.46 | 19 | -1.50 [-6.25, 3.24] | 0.533 |
| 3-month f-u | 26.62 | 11.99 | 25 | 26.66 | 14.01 | 16 | -0.03 [-5.33, 5.27] | 0.990 |
| 6-month f-u | 25.58 | 12.91 | 22 | 25.45 | 14.69 | 15 | 0.12 [-5.5, 5.75] | 0.966 |

^a^ Estimated means are presented in this table.

^b^ f-u : follow-up

SIAS, Social Interaction Anxiety Scale; SPIN, Social Phobia Inventory; BSPS, Brief Social Phobia Scale.

**Exploratory statistical analyses**

For primary outcome analyses, we have conducted additional exploratory analyses to control for the severity of positive symptoms, negative symptoms and number of sessions completed. None of these covariates were significant except for the severity of positive symptoms (F(1, 205.20)= 10.136, p=.002). Main effects investigated in LMM analyses did not change after adding these covariates as both randomized groups kept exhibiting significant improvement over time on the composite score of SA (p<.001) and no significant interaction (p>.820) nor group differences (p>.669) were observed.
